# The effects of attractive vs. repulsive instructional cuing on balance performance

**DOI:** 10.1186/s12984-016-0131-z

**Published:** 2016-03-16

**Authors:** Catherine Kinnaird, Jaehong Lee, Wendy J. Carender, Mohammed Kabeto, Bernard Martin, Kathleen H. Sienko

**Affiliations:** Department of Mechanical Engineering, University of Michigan, G.G. Brown Laboratory, 2350 Hayward St., 48109 Ann Arbor, MI USA; Department of Otolaryngology, University of Michigan, 1500 E Medical Center Dr., 48109 Ann Arbor, MI USA; Department of Internal Medicine, University of Michigan, 1500 East Medical Center Dr., 48109 Ann Arbor, MI USA; Department of Industrial & Operations Engineering, University of Michigan, 1205 Beal Ave, 48109 Ann Arbor, MI USA

## Abstract

**Background:**

Torso-based vibrotactile feedback has been shown to improve postural performance during quiet and perturbed stance in healthy young and older adults and individuals with balance impairments. These systems typically include tactors distributed around the torso that are activated when body motion exceeds a predefined threshold. Users are instructed to “move away from the vibration”. However, recent studies have shown that in the absence of instructions, vibrotactile stimulation induces small (~1°) non-volitional responses in the direction of its application location. It was hypothesized that an attractive cuing strategy (i.e., “move toward the vibration”) could improve postural performance by leveraging this natural tendency.

**Findings:**

Eight healthy older adults participated in two non-consecutive days of computerized dynamic posturography testing while wearing a vibrotactile feedback system comprised of an inertial measurement unit and four tactors that were activated in pairs when body motion exceeded 1° anteriorly or posteriorly. A crossover design was used. On each day participants performed 24 repetitions of Sensory Organization Test condition 5 (SOT5), three repetitions each of SOT 1–6, three repetitions of the Motor Control Test, and five repetitions of the Adaptation Test. Performance metrics included A/P RMS, Time-in-zone and 95 % CI Ellipse. Performance improved with both cuing strategies but participants performed better when using repulsive cues. However, the rate of improvement was greater for attractive versus repulsive cuing.

**Conclusions:**

The results suggest that when the cutaneous signal is interpreted as an alarm, cognition overrides sensory information. Furthermore, although repulsive cues resulted in better performance, attractive cues may be as good, if not better, than repulsive cues following extended training.

## Introduction

Vibrotactile feedback has been shown to improve postural performance during quiet and perturbed stance in healthy young adults [1–4], older adults [1, 5, 6] and individuals with balance impairments [6–10]. Vibrotactile displays for balance-related applications typically include an array of tactors distributed around the head [11, 12] or the torso [9, 13, 14]. For torso-based systems, an inertial measurement unit (IMU) measures torso linear acceleration(s) and/or angular velocity(ies) and tactors are activated when body motion exceeds predefined thresholds in either the anterior/posterior (A/P) and/or medial/lateral (M/L) directions. The feedback is typically associated with the specific instruction to “move away from the vibration” (i.e., repulsive cuing strategy) [2, 15–17]. These displays serve as “alarm” signals to indicate body movement away from a stable posture, and require volitional postural responses [18]. However, volitional postural responses to alarm signals may be incongruent with the kinesthetic messages from the tactile receptors stimulated. Recent studies have shown that in the absence of instruction, vibrotactile stimulation induces small (~1°) non-volitional responses in the direction of its application location when vibrations are applied to the skin over the internal oblique (IO) and erector spinae (ES) muscle locations [18–20]. Therefore, we hypothesize that an attractive cuing strategy (i.e., “move toward the vibration”) leveraging this “natural” tendency may further facilitate the use of vibrotactile feedback for balance related applications. Attractive cuing has previously been used during walking [21, 22], driving [23] and flying [24] applications. The purpose of this study was to characterize the effects of cuing strategy on postural performance.

## Methods

**Fig. 1:**
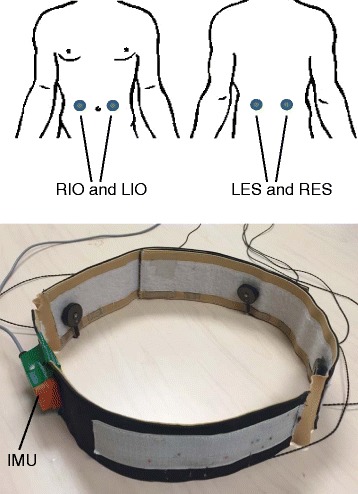
*Top*: Illustration of tactor placement on the torso over the areas corresponding to the *left* and *right* internal oblique (LIO, RIO) muscles and *left* and *right* erector spinae (LES, RES) muscles. *Bottom*: Photograph of the belt, IMU, and tactors

Eight healthy older adults (5 F, 3 M, 65 ± 2 years) were recruited to participate in two non-consecutive days of computerized dynamic posturography testing (Equitest, Neurocom Inc). This study was reviewed and approved by the University of Michigan Internal Review Board and all subjects gave written informed consent in accordance with the Declaration of Helsinki. Subjects wore a polyester t-shirt and thin athletic socks. Four tactors (C-2, EAI Inc) were mounted in a belt with Velcro and placed over the shirt on the torso over the right and left internal oblique (front) and right and left erector spinae (back) locations (Fig. 1) at the level of the L4/L5 segment [18]. The tactors were activated in pairs (IO or ES) when the torso tilt and half the tilt rate (P + 0.5D), measured by a six degree-of-freedom IMU (MTx, XSens Inc) placed at the L3 vertebrae and sampled at 80Hz, exceeded a 1° threshold in either the anterior or posterior directions. A crossover design was used wherein half of the subjects were given repulsive cues on day 1 (move away from the vibration) and the other half of the subjects were given attractive cues on day 1 (move toward the vibration). Testing days were separated by at least one day, but were no more than one week apart.

Prior to testing, subjects were given a minimum of 5–10 min, but not more than 15 min, of active training on how to interpret and respond to the vibrotactile feedback during balance testing. Participants were told to “initiate a postural correction (toward or away) when you feel the vibration”. Participants performed the same set of tasks on each testing day. The experimental protocol included three distinct parts: 1) 24 repetitions of Sensory Organization Test (SOT) condition 5 (sway referenced support surface, eyes closed); 2) three repetitions each of SOT 1 – 6; 3) three repetitions the Motor Control Test (MCT with large back translations, eyes closed) and five repetitions of the Adaptation Test (ADT with large toes up rotations, eyes closed). Data from parts 1 and 3 are included in the present study.

Performance metrics included root mean square (RMS) sway in the A/P direction (A/P RMS), percentage time during each trial within a 1° no feedback zone (Time-in-Zone), and a 95^th^ percentile confidence interval ellipse fit to the sway data (95 % CI Ellipse). For the ADT and MCT tests additional performance metrics included maximum A/P tilts and the percentage of trials in which subjects returned to the no feedback zone after the perturbation within the collected time.  Cuing strategy preferences were collected using a questionnaire.

Data were processed using Matlab (Mathworks, Natick, MA). IMU raw data were low-pass filtered using a 2^nd^ order Butterworth filter with a cutoff of 10 Hz [25]. The natural log of all metrics was used to ensure normal distributions. Since there were multiple outcomes per participant, a mixed effect model with a random intercept was used to determine the effects of repulsive and attractive cuing strategy, and the carry-over effects between testing days. The learning effects across the 24 SOT trials were compared by performing a regression analysis on the logarithmically transformed data and examining the interaction effects between trial and cuing strategy. The mixed effect analyses were performed using Stata 13.1 (StataCorp 2013).

At the end of the second testing day user preferences were collected with a six-question survey. Questions included two Likert-style questions (e.g., “I found repulsive cues easy to use”) and four fill in the blank questions (e.g., “I performed the balance task the best with ____ cuing”).

## Results

**Fig. 2:**
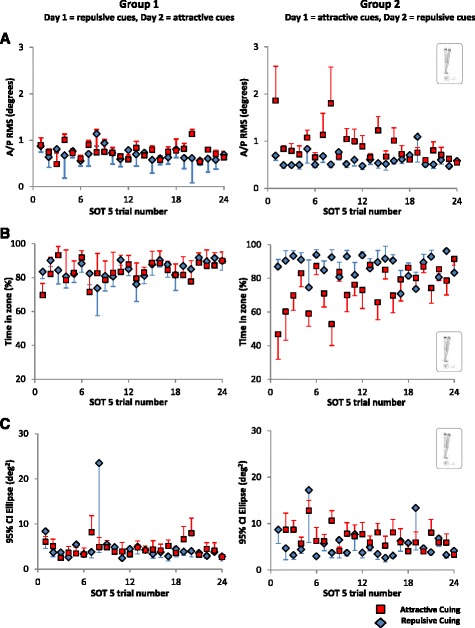
Group averages for each trial of SOT 5 for repulsive (*blue*) and attractive (*red*) cuing (±SE) for (**a**) A/P RMS tilt, (**b**) Time-in-zone and (**c**) 95 % CI Ellipse fit to the A/P vs M/L tilts

According to all metrics, postural performance for SOT 5 was better with repulsive cuing compared to attractive cuing (Fig. 2). Sway in the A/P direction (A/P RMS) was significantly smaller (~1°) for repulsive versus attractive cues (*p* < 0.001); A/P RMS values were also significantly smaller (~1°) on Day 2 versus Day 1 (*p* < 0.001) with minimal carry over effects between the two days (*p* = 0.9). The 95 % CI Ellipse values were significantly smaller (~1 deg^2^) for repulsive versus attractive cues (*p* = 0.004) and significantly smaller (~1 deg^2^) on Day 2 versus Day 1 (*p* = 0.002) with minimal carry over effects (*p* = 0.2). Subjects spent slightly more time (~1 %) in the no feedback zone (Time-in-Zone) when using repulsive cues (*p* < 0.001) and slightly more time (~1 %) in the no feedback zone on Day 2 compared to Day 1 (*p* = 0.003) with minimal carry over effects (*p* = 0.6). The slope of the regression analysis fit to the logarithmically transformed A/P RMS data (not shown) was steeper (i.e., more negative) for attractive versus repulsive cues, however it was not significant (*p* = 0.2). The slope of the fit to the logarithmically transformed Time-in-Zone data (not shown) was steeper (i.e., more positive) for attractive versus repulsive cues (*p* = 0.002). The slopes of the fits to the logarithmically transformed A/P RMS and 95 % CI Ellipse data significantly decreased as a function of trial (*p* = 0.001) regardless of day or cuing strategy (no interaction effects).

**Fig. 3:**
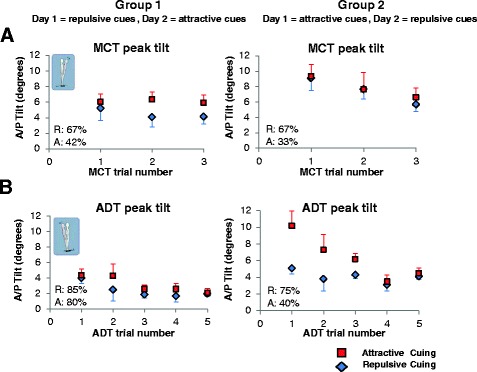
Group averages of peak A/P tilts for (**a**) MCT and (**b**) ADT trials for repulsive (*blue*) and attractive (*red*) cuing (±SE). Percentages on graphs indicate percent of trials in which subjects returned to the no-feedback zone after perturbation within the collected time period for repulsive and attractive cuing

Peak A/P tilts for the MCT and ADT perturbations (Fig. 3) were significantly smaller for all conditions with repulsive cuing (*p* = 0.001). Also, the percentage of trials in which subjects returned to the no feedback zone after the perturbation was significantly larger for repulsive cuing versus attractive cuing (Fig 3, *p* = 0.01).

From the survey questionnaire, five subjects preferred repulsive cuing and two preferred attractive cuing (one had no preference) and all eight subjects said that they learned to use repulsive cuing the quickest.

## Discussion

Performance improved with both cuing strategies for all conditions (SOT, MCT, ADT); however, participants performed better when using repulsive cues regardless of whether they completed repulsive cuing trials on the first or second day of testing. The regression analysis demonstrated that participants’ rates of improvement were greater for attractive versus repulsive cuing for the A/P RMS sway and Time-in-Zone metrics.

The results do not fully support our initial hypothesis based on the benefit of congruence between sensory and cognitive information. It appears that when a sensory signal is attributed an “alarm” significance, the corresponding messages are interpreted according to the cognitive assignation and not as the natural feedback. This suggests that avoidance behavior, derived from life experience and/or default sensorimotor pathways as in the withdrawal reflex, prevail over apparently conflicting sensory information. It may be hypothesized that the cognitive system resolves a possible conflict by choosing a low cost (known) solution that may not be optimal in terms of response time. The lower initial performance with attractive cuing, particularly when it is presented on Day 1 (Group 2) may be indicative of a cognitive “incongruence” engendered by the less natural/common requirement of attraction toward a perturbation. Although in the end both strategies led to a similar level of performance, the findings would tend to support the use of a repulsive cuing strategy when time to learn to use a torso-based vibrotactile feedback system is limited. However, based on the steeper slopes of the fits to the logarithmically transformed SOT 5 data (not shown in the figures), performance using attractive cues following a training period may be as good, if not better, than performance using repulsive cues. Future work should involve extended within and across session training protocols.

## Abbreviations

A/P: anterior/posterior
ADT: adaptation test
CI: confidence interval
ES: erector spinae
IO: internal oblique
IMU: inertial measurement unit
MCT: motor control test
M/L: medial/lateral
RMS: root mean square
SOT: sensory organization test
